# Local and systemic immunological response in feline chronic gingivostomatitis: a critical review

**DOI:** 10.3389/fimmu.2025.1572631

**Published:** 2025-09-11

**Authors:** Rafael S. Lopes, Pedro P. Carvalho, Maria A. Pires, Paulo Rodrigues-Santos, Eduardo Costa, João F. Requicha

**Affiliations:** ^1^ Department of Veterinary Sciences, University of Trás-os-Montes e Alto Douro (UTAD), Vila Real, Portugal; ^2^ Vasco da Gama Research Center (CIVG), University School Vasco da Gama (EUVG), Campus Universitário de Lordemão, Coimbra, Portugal; ^3^ Animal and Veterinary Research Center (CECAV) - Associate Laboratory for Animal and Veterinary Science (AL4Animals), UTAD, Vila Real, Portugal; ^4^ Vetherapy, San Francisco, CA, United States; ^5^ Faculty of Medicine (FMUC), Institute of Immunology, University of Coimbra, Coimbra, Portugal; ^6^ Center for Neurosciences and Cell Biology (CNC), Laboratory of Immunology and Oncology, University of Coimbra, Coimbra, Portugal; ^7^ Center for Investigation in Environment, Genetics and Oncobiology (CIMAGO), University of Coimbra, Coimbra, Portugal; ^8^ Coimbra Institute for Clinical and Biomedical Research (iCBR), University of Coimbra, Coimbra, Portugal; ^9^ Center for Innovation in Biomedicine and Biotechnology (CIBB), University of Coimbra, Coimbra, Portugal; ^10^ Clinical and Academic Center of Coimbra (CACC), Coimbra, Portugal; ^11^ ⁠Institute of Experimental Pathology, Faculty of Medicine, Azinhaga de Santa Comba, Universidade de Coimbra, Coimbra, Portugal; ^12^ ⁠CQC-IMS, Chemistry Department, University of Coimbra, Coimbra, Portugal

**Keywords:** cat, feline chronic gingivostomatitis, oral cavity, immunologic response, oral *lichen planus*

## Abstract

**Introduction:**

A comprehensive understanding of the oral immune response in feline chronic gingivostomatitis (FCGS) is crucial for veterinarians to improve clinical and therapeutic decisions. This critical review addresses the local and systemic immune responses associated with FCGS.

**Methods:**

A comprehensive database search was conducted using the PubMed/MEDLINE database, resulting in 3,358 studies. Following a rigorous screening process, in accordance with the Preferred Reporting Items for Systematic Reviews and Meta-Analyses (PRISMA) guidelines, 17 publications were included in this review.

**Results:**

The local immune response in FCGS is primarily evaluated through histopathological and immunohistochemical analyses of oral biopsy samples, and by analysis of saliva. Histopathological analysis reveals a dense lymphoplasmacytic infiltration within the mucosa, indicating chronic inflammation. Immunohistochemical staining identifies increased numbers of mast cells, altered expression of immunoglobulins (IgG, IgM, and IgA) and presence of immunomarkers (CD3+, CD4+, CD8+ T cells and Mott cells), indicating immune dysregulation. Systemic immune features of FCGS are investigated in blood samples through flow cytometry, polymerase chain reaction, and enzyme-linked immunosorbent assay. A consistent finding is an increased level of pro-inflammatory cytokines (IL-6, TNF-α, and IFN-γ), indicating intense systemic immune activation. Neutrophilia, disrupted CD4/CD8 T-cell ratio, a reduction in CD21+ B cells and alterations in regulatory T cells expressing FOXP3, suggests chronic immune regulation dysfunction.

**Discussion:**

The findings highlight a complex relationship between local and systemic immune responses, by significant alterations in T cell subsets, pro-inflammatory cytokines, and immunoglobulin expression. The frequent presence of CD3+, CD4+, and CD8+ T cells, along with impaired regulatory T cell (FOXP3+) function, suggests that dysregulated cell-mediated immunity is a key factor in the pathogenesis of FCGS. Elevated systemic immunomarkers (IL-6, TNF-α, and IFN-γ), provide further evidence of a chronic immune activation state. The immunopathological similarities observed between FCGS and human oral lichen planus reinforce the potential of FCGS individuals as a spontaneous model for comparative research. This review found that there is a lack of comprehensive information on the oral immune response of FCGS. Further observational and experimental studies focusing on the link between local and systemic immune responses are essential to fully understand the complexity and guide the development of novel, evidence-based therapeutic strategies.

## 1 Introduction

The mucosal immune system is a localized and specialized immune network that protects the inner surface of the human body, encompassing nearly the entire human body. It encompasses the mucosal surface of the oral-pharyngeal cavity, gastrointestinal (GI) tract, respiratory tract, urogenital tract, and the exocrine glands. Despite their distinct locations, the mucosal immune systems of different organs share analogous anatomical organization and characteristics. Given the more extensive understanding of the GI mucosal immune system, this discussion will focus on the features of the mucosal immune system based on our knowledge of the GI immune system (1).

The oral-pharyngeal cavity mucosa is constantly stimulated by antigens derived from multitude of sources, encompassing both external and internal factors including air and food antigens, among others. Although the oral mucosa forms a mechanical, thick and dense barrier, due to its histological particularities, the periodontal epithelial zone is a fragile region for the entry of microorganisms. Therefore, a robust local immune system is crucial for the maintenance of the oral mucosa integrity and tolerance, where the presence of T lymphocytes plays a critical role (1, 2).

Feline chronic gingivostomatitis (FCGS) is a common inflammatory disease, being estimated to affect between 0.7% and 10-12% of cats under veterinary observation. FCGS is associated to immunodeficiency, dysregulation, or loss of immunotolerance of the oral immune system (1, 3–6).

The disease is characterized by chronic clinical signals with oral pain, weight and body condition loss, inappetence, ptyalism, dysphagia, reduced grooming, hypersalivation, halitosis and hyporexia or anorexia (3–5, 7–10).

The typical macroscopic presentation of FCGS involves inflammatory lesions in the oral mucosa extending across the mucogingival junction, allowing differentiation from gingivitis. Despite significant advances in diagnostic and therapeutic methods, the survival rate of cats affected with FCGS has not improved in recent decades. These animals have a poor quality of life, and some owners choose to practice euthanasia (6).

FCGS is an immunopathogenic disease that shares many similarities with oral *lichen planus* (OLP) in humans. Emerging evidence suggests that FCGS may serve as a relevant spontaneous model due to key immunopathological and clinical parallels. Both diseases are characterized by chronic, immune-mediated inflammation of the oral mucosa, with histopathological features dominated by dense lymphoplasmacytic infiltrates and basal cell degeneration. In both FCGS and OLP, CD3+ T lymphocytes, particularly CD4+ helper and CD8+ cytotoxic subsets, are prevalent in lesional tissues. This finding supports the hypothesis of a T-cell-mediated mechanism of epithelial damage (3, 11–14). Elevated levels of pro-inflammatory cytokines, such as interleukin-6 (IL-6), tumor necrosis factor-alpha (TNF-α), and interferon-gamma (IFN-γ), have been documented in both conditions. These cytokines contribute to persistent immune activation and tissue remodeling.

Clinically, both diseases follow a chronic relapsing course, are often refractory to conventional treatments, and may require immunosuppressive or immune-modulating therapies. The clinical, histopathological and immunological features described here suggest that FCGS offers translational potential as a naturally occurring animal model for studying the pathogenesis and therapeutic responses of OLP. Further comparative investigations could enhance the utility of FCGS in modelling human oral mucosal autoimmune diseases (15–17).

Despite extensive scientific research on this topic, the etiology of the FCGS remains unclear and poorly understood.

The present study aims to provide a critical and comprehensive review of the available literature on the subject, with a view to clarifying the local and systemic immunological responses involved in FCGS, thus contributing to a more precise characterization of the disease. The consolidation of current knowledge may support the development of more effective therapeutic strategies for clinical application.

## 2 Materials and methods

This critical review was conducted in accordance with the 2020 Preferred Reporting Items for Systematic Reviews and Meta-Analyses (PRISMA) guidelines and the recommendations of the Cochrane Collaboration (Cochrane Handbook for Systematic Reviews of Interventions) (18).

### 2.1 Search protocol

The eligibility criteria described above were used to develop specific search strategies for databases. In April of 2024, a search was performed at Pubmed/MEDLINE’s database for published and in publication records, in order to select all articles potentially relevant to the question of this investigation.

A list of controlled medical subject headings (MeSH) words was used mixed with Boolean operator words “AND” or “OR”, as conjunctions to combine or exclude keywords resulting in more focused and productive results. The MeSH words used are “Immunology OR inflammation OR cells AND “oral cavity” OR mouth OR tongue OR lingual OR “oral mucosa” AND feline”.

This type of structuring and terminology allows the differences of the indexing processes in bibliographic databases to be minimized, guaranteeing a greater number of obtained references.

### 2.2 Study selection

Following the removal of duplicates, two reviewers independently screened 3,361 titles and abstracts to identify studies that met the inclusion criteria. Abstracts which did not meet the eligibility criteria were excluded at this stage.

For the remaining studies, the same reviewers conducted a comprehensive assessment of the full texts to determine their suitability for inclusion in the critical review, in accordance with the established eligibility criteria. Further relevant references were searched for in the bibliographic reference list of the selected studies. This study considered participant and population eligibility, exposure, outcome, and context.

The studies were selected based on the following inclusion criteria: (i) FCGS in domestic cats (*Felis catus*), (ii) identification of local and/or systemic characterization of the disease and (iii) publication in English, French, Spanish or Portuguese.

Studies were excluded if they were not published in the English or another previous specified language, if they were dissertations, theses or review articles, if they included oral conditions other than FCGS, if they focused exclusively on treatment, or if the full text was not available online. Regarding the latter criterion, a total of 38 studies were excluded due to unavailability of the full text through academic databases or institutional subscriptions. Despite attempts to obtain these documents via direct contact with the corresponding authors, these efforts were unsuccessful, and the studies were consequently excluded from analysis.

### 2.3 Data collection

After applying all exclusion criteria and critically evaluating, the final list of selected studies was determined. Each study was evaluated descriptively and the data obtained were summarized in a Microsoft® Excel® database which was adapted to the research containing the indicative elements of the study: author, year of publication and geographic location, study design, sample size, age, breed, comorbidities, information about potential etiological agents: bacterial, fungal and viral infection, disease clinical presentation, local and systemically characterization, and methods and used immune parameters in each characterization. The animal’s age was divided in four life stages: kitten: 0-1year, young adult: 1–6 years, mature adult: 7–10 years, senior: >10 years (19).

## 3 Results

**Figure 1:**
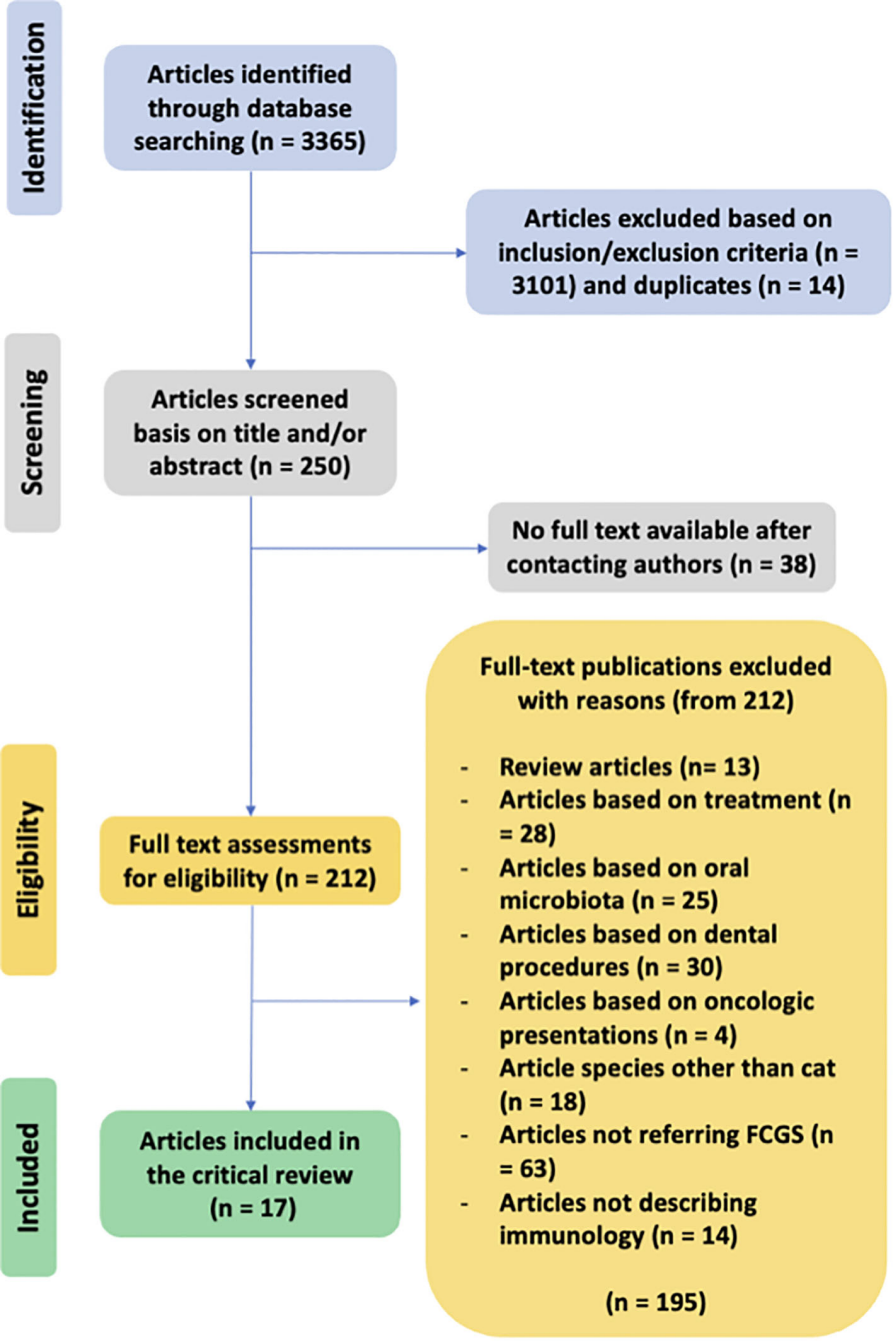
PRISMA flow chart for study selection.

The PRISMA flow chart for the selected articles used in this review can be seen in **Figure 1**. The initial search in the PubMed/MEDLINE database found a total of 3,365 abstracts.

After screening the title and abstract, 3,115 were excluded, including 14 duplicates. A total of 243 articles were selected for final full-text reading/review as they were considered potentially relevant to the study. Despite contacting the authors of 38 of the articles in question, the requested material could not be obtained. The remaining 205 articles about cats with FCGS include studies with various primary objectives. These include studies that did not focus on the immunological characterization of the disease, studies that investigated therapeutic and surgical options, others that examined the prevalence or association with comorbidities, and studies about oral/dental diseases other than FCGS.

After full reading of the 212 selected studies, seventeen of them satisfied all the inclusion criteria and were considered in this critical review. One hundred and ninety-five studies were excluded for the following reasons: (i) review articles (n=13), (ii) articles based on treatment (n=28), (iii) articles based on oral microbiota (n=25), articles based on dental procedures to treat the disease (n=30), (iv) articles referring to feline oral neoplasia (n=4), (v) articles based on species other than cat (n=18), (vi) articles not referring FCGS (n=63) and (vii) articles not describing immune characterization of the disease (n=14).

The collected data from the included 17 studies are summarized in **Supplementary Tables**. Regarding the 17 articles included in this critical and qualitative review: (i) one article identified and studied the different tumors and tumors-like lesions such FCGS; (ii) one article investigated the prevalence of FCGS in first opinion veterinary practice (20); (iii) five articles studied the association between FCGS and the presence of various oral bacteria such as *Bartonella henselae* and viruses as Feline Calicivirus (FCV) and Feline Herpesvirus type 1 (FHV-1) (21–25); (iv) six studies addressed the characterization of FCGS describing the histological, immunohistochemical, cellular, molecular and genetic changes presented in this disease (3, 13, 26–29); (v) four articles focused on the characterization of the disease, but with emphasis on different types of treatment such as tooth extraction and stem cell-based therapies (4, 30–32).

The studies included in this review were conducted in the following countries: Unites States of America (n=8), United Kingdom (n=4), France (n=2), Poland, Brazil and Portugal (n=1 of each).

The seventeen studies selected for this critical review, in total, evaluated 5,663 cats. Of the total number of cases evaluated, 566 cats were diagnosed with FCGS (8.6%).

Breed was not possible to identify in 169 of the 566 cats. The remaining 350 were from pure breeds, such as Siamese (n=17), Maine Coon (n=11), Norwegian Forest (n=5), Persian (n=8), British Shorthair and Oriental (n=4), Burmese and Chartreux (n=2), Birman, Angora and Russian blue (n=1). Other non-pure breeds have been identified as Domestic Shorthair (n=311), Domestic Medium-hair (n=6), Domestic Longhair (n=4) and mixed breed (n=63). Regarding the gender of the animals, it was only possible to identify it in 444 cats (263 males and 181 females).

Five studies did not report the age of the animals, and, in the remaining twelve studies, the 330 animals were aged from 6 months to 20 years, with the following classes: less than 6 months (n=2), 7 months to 2 years (n=31), 3 to 6 years (n=65), 7 to 10 years (n=174), 11 to 14 years (n=38) and more than 15 years (n=20). The average age of both the diseased and control animals was seven years.

Of the 17 eligible articles, seven (41%) described cases of FCGS associated with periodontal disease (n=84 animals), tooth resorption (n=32), presence of roots remnants (n=2), oral squamous cell carcinoma (SCC) (n=1), and chronic kidney disease (CKD) (n=1).

Six studies (35%) also report the presence of bacterial agents from the genus *Pasteurella*, *Pseudomonas*, *Bartonella* and *Tannarella*, and viral agents such as FCV, FHV-1, feline immunodeficiency virus (FIV) and feline leukemia virus (FeLV).

Fifty percent of these studies detected retroviruses in diseased animals by chromatographic immunoassay for FeLV antibodies (n=3) and FIV antigens by polymerase chain reaction (PCR) (n=1). Most authors used oral swabs (n=4) to identify FCV infection using PCR techniques. One study used the anti-FCV1–43 antibody in oral mucosa samples collected by incisional biopsies, and other used the cytopathic effect test (CPE). In the case of the CPE test, a swab sample was inoculated into a feline embryonic fibroblast cell line, and the cells were subsequently subjected to daily observation until the appearance of characteristic cytopathic effects (21). One article described the identification of FCV in serum samples using RT-PCR and PCR. Finally, only one study reported, by PCR on serum samples, FHV infection.

In two studies, bacterial identification was performed, one by genetic sequencing (23), and other by enzyme-linked immunosorbent (ELISA) and western blotting (24). The samples used were obtained from subjects who tested negative for both FIV and FeLV. No diagnostic tests were performed in the remaining two papers for infectious disease detection.

One study described the transcriptomic gene profile in FCGS oral mucosa tissues related to the immune and inflammatory response and pathways influenced by cytokines such as IL-6, IL-17 and IFN. Another study quantitatively compares the levels of different types of immunoglobulins (IgA, IgG and IgM) between saliva and serum in diseased cats.

### 3.1 Diagnostic approach to FCGS

The diagnostic approach to FCGS is a topic under wide discussion among specialists. There has been an effort to get detailed descriptions of the locally and systemically presentations.

This review revealed that, of the 17 selected articles, 88% (n=15) described the local presentation, while 29% (n=5) were also focused on the systemic manifestation of the disease.

In 11 studies (64.7%), the diagnose of the FCGS was based on the typical clinical presentation, and, in 13 studies (76.4%), was supported by histopathology of oral mucosa, being complemented by immunohistochemistry (IHC) assay in 47% (n=8) of studies. The studies indicate that manifestations are mostly located in the caudal areas of the oral cavity (caudal stomatitis) and present a proliferative form compared to the rostral (alveolar mucositis) presentation. It is important to note that this information is based solely on clinical presentation and features. The most prevalent inflammatory infiltrate is lymphoplasmacytic, as determined by histopathology (11, 33). Ulcerative manifestation of the disease was mostly associated with infection by FCV, FIV, FeLV, FHV or anaerobic bacteria (20).

This condition is not fully understood, but it is believed to be caused by a combination of factors including viral and bacterial infections, immune system dysfunction, and genetic predisposition (13, 23, 34). Diseased cats show distinct variations in cytokine expression and immunoglobulin profiles compared to healthy cats. These differences may contribute to inflammation in the oral cavity, which can result from unrelated health problems (3, 35, 36). In cats, gingival hyperplasia is less prevalent than in dogs. In all cases, it is accompanied by lymphoplasmacytic inflammation of varying degrees, which indicates a primary inflammatory origin (36).

### 3.2 Histopathological features of chronic gingivostomatitis

In FCGS animals, the inflammatory infiltrate was mainly composed of lymphocytes and plasma cells, with fewer neutrophils and macrophages. Quantitative analysis showed a significant increase in the number of mast cells per mm^2^ in FCGS animals compared to specific pathogen-free animals. There was a notable increase in the inflammatory cell infiltrate (inflammatory score) observed between the FCGS and periodontitis animals (26, 27, 31).

Six studies described the histopathological changes observed by biopsy in the disease of alveolar mucosa or palatoglossal arch. The mucosal epithelial changes include hyperplasia (4–15 layers of keratinocytes), parakeratosis, and vacuolar degeneration with deep multifocal epithelial ulcerations extending into the stroma. A rich infiltrate of inflammatory cells in the mucosa was also described. The observed inflammatory cells comprised a predominance of lymphocytes, plasma cells and neutrophils (13, 27). Mast cells, macrophages and occasional Mott cells were also present, within the multiple rete pegs and extending deep into the subjacent stroma (17, 21, 28). Neutrophils were predominantly observed in areas exhibiting mucosal ulceration. The studies reviewed did not describe the presence of other cell types, such as basophils or eosinophils (13).

The submucosal layer exhibited the presence of macrophages and polymorphonuclear neutrophils. Infiltrates were observed, which were either focal or multifocal and consisted mainly of plasma cells, Mott cells and varying amounts of lymphocytes, mast cells and neutrophils, especially in in the submucosa ulcerated areas. The infiltrate was mainly located near vessels and salivary gland ducts. Mast cells were predominantly located in the submucosa, and in rete cones areas, extending deeply into the submucosa. The submucosal capillaries were congested and lined with endothelial cells that bulged (13, 26, 27, 30, 31, 38).

### 3.3 Local immunological features of feline chronic gingivostomatitis

Conventional mucosal T cells (CD3+ T cells) can be classified as either major histocompatibility class II (MHC II) restricted and αβ T cell receptor (TCR)-expressing CD4+ T cells (helper T cells, Th cells) or MHC I-restricted and αβ TCR-expressing αβ T cells. These T cells develop in the thymus and subsequently migrate to mucosal sites of action following the encounter with antigenic stimuli in lymphoid tissues. However, within the epithelial layer of the mucosa, there is another unique subset of T cells. These T cells express either αβ or γδ TCRs and predominantly express CD8α homodimers, with the exception of CD8αβ heterodimers (2, 39–41).

The immunohistochemical characterization was described by six authors who identified the presence of various biomarkers in different tissue layers (**Supplementary Table 1**). CD3+ T cells were observed in the epithelial and subepithelial layers, while B cells and Mott cells were restricted to the submucosal and subepithelial stroma. CD4 and CD8, T subsets immunoreactive cells followed the general distribution pattern of T lymphocytes, mainly located in the mucosal and submucosal layers. The superficial mucosa showed equal distribution of FOXP3+ cells and CD25+ cells (3, 13, 31, 32, 37, 38).

The severity of clinical lesions was differentiated by one author into lower and higher grades based on microscopic observation of tissue inflammation. The score was categorized on a four-point scale: normal (0), mild inflammation (1), moderate inflammation (2), and severe inflammation (3). In sections graded 0 or 1, only occasional CD3+ T cells were observed in intraepithelial and subepithelial areas, as well as around capillaries and salivary ducts within the lamina propria and submucosa. Sparse CD79α+ B cells were also observed, predominantly intracytoplasmic. In samples with high macroscopic changes (grade 2 and 3), dense infiltrates of CD3+ T cells were observed in the lamina propria underlying areas where the epithelium shows marked degenerative changes. These cells sometimes form a prominent ‘lichenoid band’ on the surface of the lamina propria (3).

The CD3 lymphocyte population showed a predominance of CD8+ cells in the subepithelial and intraepithelial layer. CD79α+ B cells were identified in the submucosal and lamina propria layers, consistent with B cells and plasma cells. These cells were also observed surrounding blood vessels, salivary ducts, and skeletal muscle fibers in the lamina propria and deeper submucosa layer. The localization of IgG+ plasma cells commonly corresponds to the areas where CD79α+ B cells were found. Occasionally, small numbers of IgA+ plasma cells were found near the basement membrane in the subepithelial layer. Usually, plasma cells IgM+ are sparse. Leucocyte antigen L1+ (L1) cells were identified in the lamina propria and submucosa with epithelial degeneration, more expressive in grades 2 and 3, and rare in grades 0 and 1. In some sections, the L1+ cells were concentrated in the superficial lamina propria and around deeper blood vessels. In sections with high levels of inflammatory cell infiltration, L1+ cells were also found in the connective tissue stroma surrounding bundles of skeletal muscle fibers and salivary glands (3, 35, 42).

A correlation was observed between the severity of mucosal tissue inflammation and the extent of MHC II labelling. In tissues with severity scores of 0 or 1, MHC II+ cells, exhibiting dendritic or spindle-shaped to mononuclear morphology, were present in small numbers within the epithelium and lamina propria/submucosa. On occasion, small, focal aggregates of these cells were observed in the lamina propria or connective tissue stroma surrounding skeletal muscle fibers. Some skeletal muscle fibers exhibited a minor granular cytoplasmic expression of MHC II. In tissues with severity scores of 2 or 3, moderately to densely packed populations of MHC II+ cells were observed, displaying a variety of morphological appearances. The MHC II-labelled cells were observed in close proximity to and infiltrating the salivary duct epithelium (3, 35).

Mikiewicz and colleagues, used CD3 and CD79α to identify T and B-cell populations within lymphoid infiltrates. However, as these biomarkers do not distinguish reactive from neoplastic lymphocytes, their diagnostic value for lymphoma is limited without complementary morphological evaluation. S100 marker was employed in a separated context to support the diagnostic of amelanotic melanoma (38, 43).

In 2020, Vapniarsky and colleagues identified 2,310 genes that showed at least a 2-fold difference in regulation between diseased and healthy cat tissues. Of these genes, 1,331 were upregulated and 979 were downregulated. Cluster analysis of these differentially expressed genes demonstrated distinct clustering between diseased and healthy tissue samples (13, 43).

Principal analysis revealed a distinct clustering pattern among genes related to the disease state, specifically showcasing a clear grouping of genes from diseased tissues. The study used UC Davis platform (Comprehensive Cancer Center Genomics Shared Resource) to analyze upregulated genes in diseased versus healthy tissues, revealing a substantial enrichment (ranging from 1.8 to 4.7-fold) of genes associated with multiple pathways. The article encompasses a multitude of subjects, including T-cell signaling, cell adhesion molecules, leukocyte trans-endothelial migration, NF-kappa B signaling pathways, extracellular matrix-receptor interactions, cytokine-cytokine receptor interactions, complement and coagulation cascades, Fc-gamma mediated phagocytosis, and natural killer cell-mediated cytotoxicity. In contrast, the analysis of downregulated genes using UC David’s platform revealed a 1.5 to 3.1-fold enrichment of genes associated with pathways such as axon guidance, metabolic pathways, rap1 signaling pathway, hippo signaling pathway, and tight junctions among others (3, 6, 13, 31, 32, 38).

**Figure 2:**
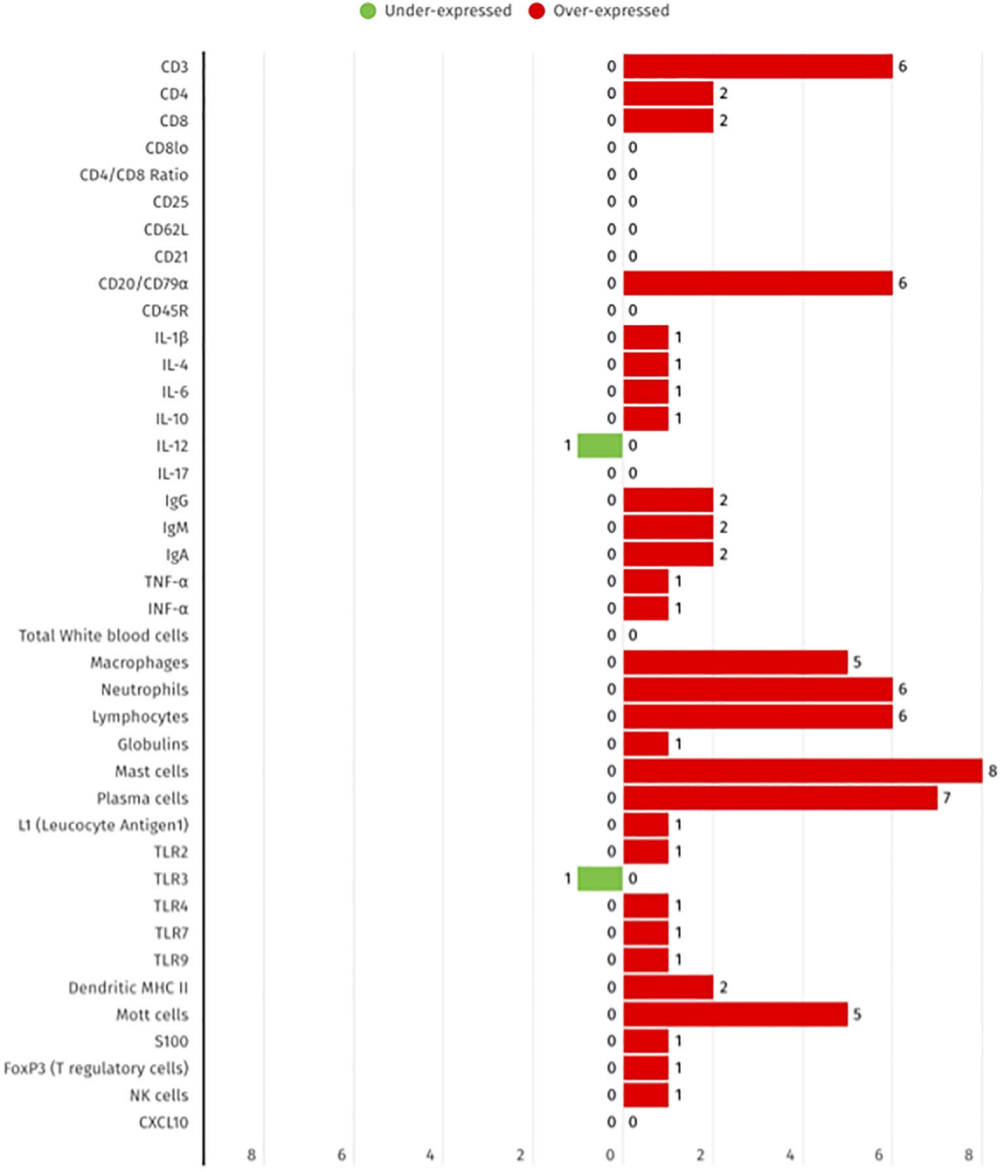
Summary of the differential expression of local immune biomarkers in FCGS animals, according to the 17 selected studies. The red bars show the number of studies that report biomarker overexpression, while green bars represent the number of studies that represent under expression.

In accordance with the 17 studies selected for inclusion in this review, it was observed that over-expression was predominantly reported across various immune markers, including plasma cells (8 studies), CD3+ T cells (6), and neutrophils (6). Under-expression was less frequently observed, with TLR3 and IL-10 being the only biomarkers that were downregulated in at least one study. These findings reflect the locally enhanced immune activation and cellular infiltration that is characteristic of FCGS, as illustrated in **Figure 2** by the summarized differential expression of local biomarkers.

### 3.4 Systemic immunological features of feline chronic gingivostomatitis

Regarding the systemic presentation of the disease, there is scarce information to properly characterize its manifestation. Out of the 17 authors, only five have mentioned methods that enable a systemic characterization of FCGS, summarized in **Supplementary Table 2**.

The preferred study methods described are flow cytometry, protein electrophoresis and complete blood cell counts. Additionally, two author suggest combining these methods with ELISA and serum biochemistry. Cats with FCGS typically show signs of systemic inflammation, including a significant increase in white blood cell count due to neutrophilia, polyclonal hypergammaglobulinemia, and increased expression of proinflammatory serum cytokines. Affected cats exhibit elevated neutrophil counts exceeding the reference interval (10.7 × 10^3^ ± 4.8 × 10^3^; reference range: 2.0 × 10^3^ – 9.0 × 10^3^). Cats had both higher and lower total serum immunoglobulin levels, as well as normal percentages of circulating CD4+ T cells. However, the percentage of circulating CD8+ T cells was significantly higher than the reference interval (29% ± 18.57%; reference range: 16 ± 4.18%), resulting in a low CD4/CD8 ratio. This suggests ongoing cytotoxic immune activation (13, 32, 37).

The circulation CD8+ T cells were analyzed to identify CD8+^hi^ and CD8+^low^ populations. The majority (>80%) were CD5+ cells, confirming their identity as CD8^low^ T cells. The most notable change in lymphocyte phenotype in cats with FCGS was a significant increase in effector memory CD8+ T cells, accompanied by a reduction in central memory cells. These CD8+ T cells exhibited an activated phenotype, characterized by a higher proportion of CD25+ CD62L- cells (13, 44, 45).

Although there was no significant difference observed in the phenotype of activated CD4+ T cells, their trend closely mirrored that of activated CD8+ T cells in cases of FCGS. This suggests a parallel activation pattern in both CD4+ and CD8+ T cell populations in affected cats. Additionally, a noteworthy finding was the significant decrease in the circulating percentage of CD21+ B cells in FCGS compared to healthy control cats. The decrease in CD21+ B cells indicates a change in the B cell population, which may be a factor in the immune dysregulation observed in FCGS (13, 32, 46).

FCGS cases exhibit a notable elevation in pro-inflammatory serum cytokines such as TNF-α, IL-1b, and IFN-γ, indicating an increased inflammatory response in affected cats. Furthermore, a significant increase in FOXP3+ cells, including both CD8+ and CD4+ T cells, was observed in FCGS animals. The CD4+ T cells in healthy cats, include a subset of FOXP3+ cells which comprise approximately 6% of the total CD4+ T cells. However, in cats with FCGS, the proportion of these cells showed a significant increase ranging from 8.3% to 31.8% (6, 13, 31, 32).

**Figure 3:**
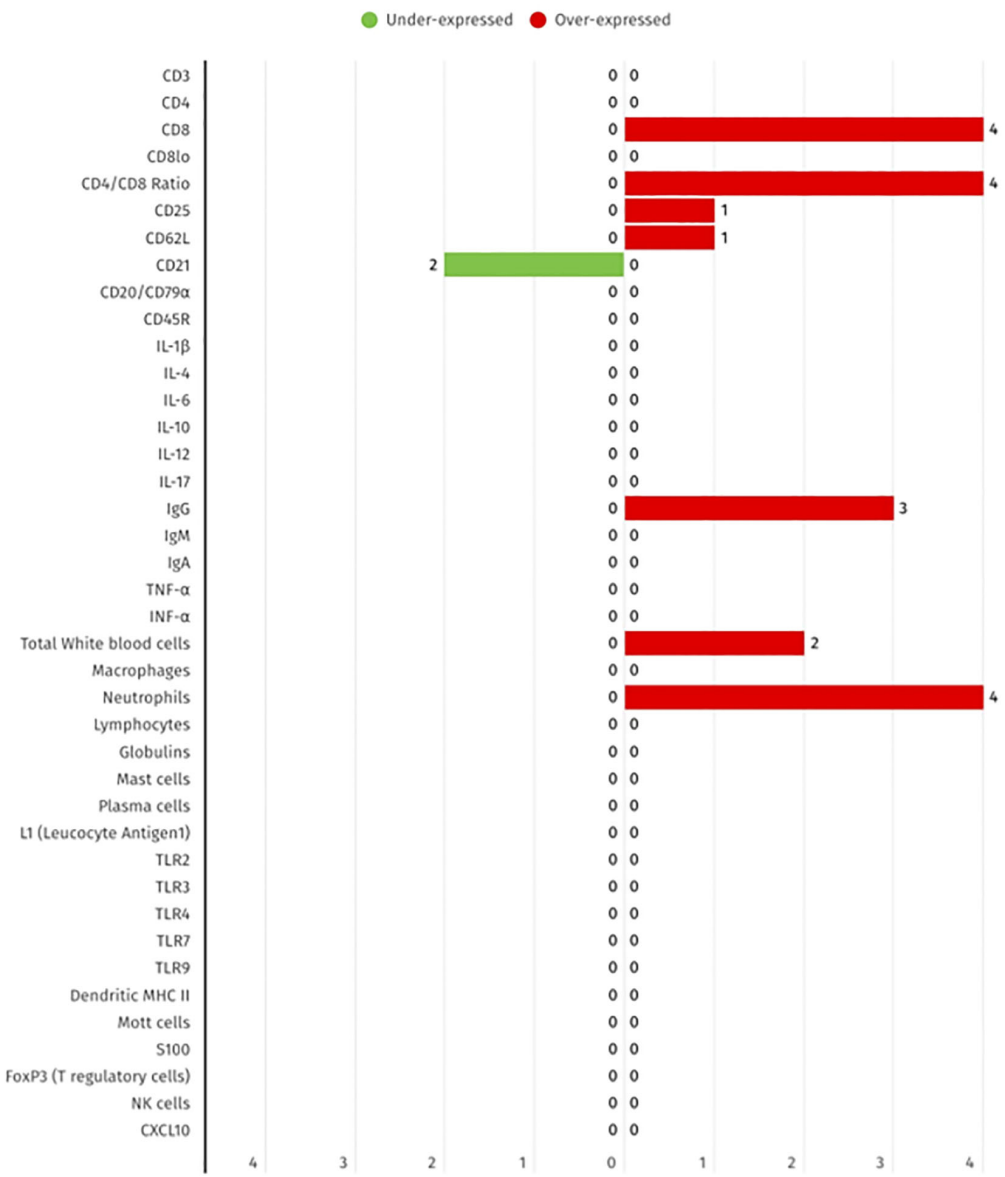
Summary of the differential expression of systemic immune biomarkers in FCGS animals, according to the 17 selected studies. The red bars show the number of studies that report biomarker overexpression, while green bar represent the number of studies that represent under expression.

As demonstrated in **Figure 3** from the selected studies, the biomarkers evaluated include cytokines, T cell subsets, B cells, and innate immune cells, measured in blood and serum. While certain subsets, such as CD8+ T cells and neutrophils, are more frequently reported as elevated, others including CD21+ B cells and the CD4/CD8 ratio are more commonly described as decreased. These findings provide further evidence to support the systemic immune dysregulation that has been previously observed in FCGS.

### 3.5 Summary of both local and systemic inflammatory biomarkers

**Figure 4:**
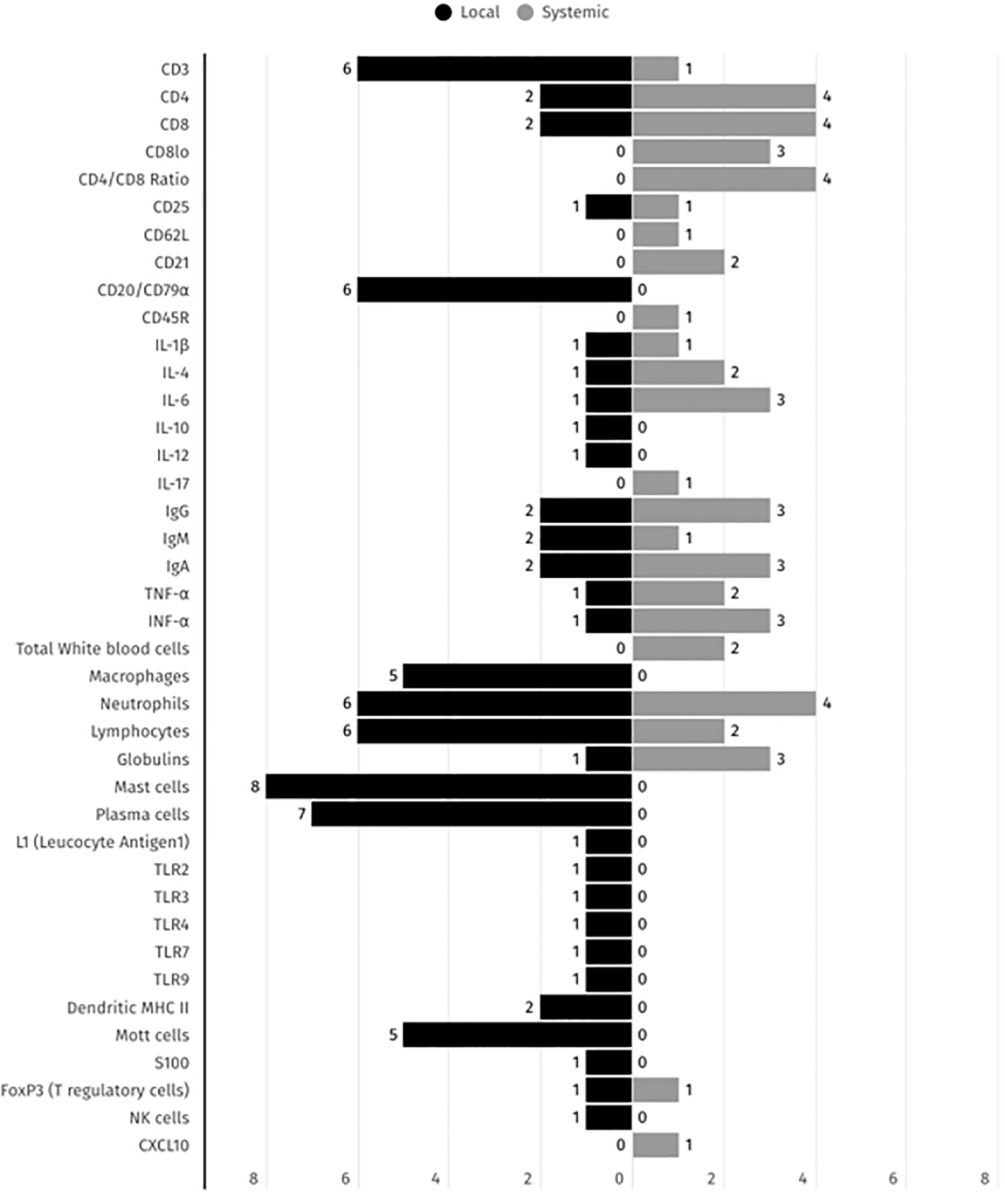
Summary of the immune biomarkers distribution across local and systemic presentations detected in FCGS animals, according to the 17 selected studies. The black and grey bars represent the number of studies reporting each immune biomarker detection in both local and systemic presentations, respectively.

Regarding the identified immune parameters, 16 were identified in local and systemic presentations including CD3, CD4, CD8, CD25, IL-1β, IL-4, IL-6, IgG, IgA, IgM, FOXP3+ (Treg cells), TNF-α, IFN-γ, lymphocytes, neutrophiles and immunoglobulins as illustrated in **Figure 4**.

CD8+ T cells present cytotoxic action capable of recognizing antigenic fragments presented by infected or cancerous cells and inducing their death through a variety of mechanisms. These include the release of cytotoxic proteins, such as perforins and granzymes, which destroy the target cell and also inducing apoptosis Furthermore, CD8+ cells play a pivotal role in the formation of immunological memory. Following the resolution of an infection, a proportion of CD8+ T cells differentiate into memory cells, enabling a more rapid and robust immune response in the event of a subsequent infection with the same agent (44).

CD4+ T cells expressing high levels of CD25, the α-chain of the interleukin-2 (IL-2) receptor, represent a heterogeneous population comprising both activated effector T cells and regulatory T cells (Tregs). In humans, the expression of CD25 alone is not sufficient to accurately identify Tregs, as it is also transiently upregulated on activated conventional T cells. The designation of CD4+CD25+CD127^low^– T cells as *bona fide* Treg cells is based on the observation that these cells exhibit high FOXP3 expression and potent immunosuppressive activity. Consequently, this characterization requires the inclusion of additional biomarkers, such as the transcription factor FOXP3, which is critical for Treg lineage commitment and function, and the low or absent expression of the IL-7 receptor α-chain (CD127). The CD4+CD25+CD127^low^ FOXP3+ phenotype is widely accepted as the most reliable signature for human Tregs. These cells are pivotal in the maintenance of peripheral immune tolerance, primarily through the suppression of effector T cell responses via cytokine-mediated mechanisms (14).

In human OLP, Treg dysregulation appears to play a significant role in disease pathogenesis. Zhou and colleagues (2016) reported an increased frequency of peripheral CD4+CD25+CD127^low^– T cells and elevated FOXP3 expression within oral lesions of patients diagnosed with OLP. Despite an increase in the number of these cells *in vitro*, they exhibited impaired suppressive function, suggesting a functional deficiency. Furthermore, a higher proportion of FOXP3+ T cells co-expressing IL-17 was detected, indicating a potential shift towards a pro-inflammatory phenotype (14, 47).

Also, in humans, the oral mucosa, epithelial cells and immune cells produce a wide range of cytokines, including IL-1β, IL-6, TNF-α, granulocyte-monocyte colony-stimulating factor (GM-CSF) and transforming growth factor-β (TGF-β), which contribute to an environment that impacts T cell activation, proliferation and differentiation. Furthermore, TGF-β stimulates the production of IgA antibodies, thus promoting the integrity and efficacy of mucosal immunity (1).

IFN-γ, a pro-inflammatory cytokine, plays a significant role as a major effector cytokine of Th1 cells in the immune response, recruiting neutrophils and enhancing the ability of the cells to recognize and phagocytose intracellular microbes. In both OLP and FCGS, the IFN-γ is present in the context of inflammatory conditions, which are understood to be dysregulated and to contribute to the perpetuation of immunopathology (14, 21, 48).

In this review, 15 exclusive local biomarkers were identified such as CD20+ and CD79α B cells, and S100 receptor in order to differentiate between lymphoma and amelanotic melanoma and to determine which category the lesion belongs to (38). In 1999, Harley and collaborators proposed that alterations in the innate immune response may play a role in the pathogenesis of FCGS. The levels of IL-2, IL-4, IL-6, IL-10 and IFN-γ mRNAs have been demonstrated to be elevated in cats with FCGS in comparison to healthy cats. This cytokine expression has been observed to shift from a Th1 response in healthy cats to a mixed Th1-Th2 response in cats with FCGS (48).

The toll-like receptors (TLRs) family is a group of pattern-recognition receptors (PRR) that are expressed on various cells of the immune system, including dendritic cells, macrophages, B cells, among others. TLRs play a foundational role in the innate immune response, recognizing microbial PAMPs (Pathogen-Associated Molecular Patterns) such as bacteria, viruses, and fungi, conserved components found in a wide variety of microorganisms (1, 21, 49–51).

When PAMPs are recognized by TLRs, a sequence of immune responses begins, which include the synthesis of pro-inflammatory cytokines, the stimulation of immune cells and the induction of co-stimulatory molecules essential for T lymphocytes activation. There are several types of TLRs, each with a specific ability to recognize different types of PAMPs (49, 52). This diversity of receptors enables the immune system to recognize a wide range of pathogens and initiate an appropriate immune response (49).

Of the eligible articles for this review, TLR2, TLR3, TLR4, TLR7 and TLR9 were identified from mucosal biopsies and mRNA levels were determined by quantitative PCR (21).

The role of TLRs is of critical importance in the organism’s defense against bacterial infections. Furthermore, they play a significant role in the development of inflammatory and autoimmune conditions when their regulation is disrupted (21, 49). In both healthy and individuals with FCGS, Dolieslager and colleagues (2012) demonstrated that oral bacteria influence the levels of cytokine mRNAs, demonstrating that a shift from a predominant Th1 response in the healthy group to a combined Th1-Th2 response in the FCGS group was observed. The study revealed an increase in the expression of IL-2, IL-4, IL-6, IL-10, IL-12 and IFN-γ mRNAs in the affected individuals (21).

The reviewed articles also identified plasma cells expressing surface immunoglobulin biomarkers for IgA and IgM, as well as distinct populations of MHC II+ immune cells. IgA+ plasma cells were predominately located near salivary ducts and within the stroma surrounding salivary glands, whereas IgM+ plasma cells were generally sparce in these regions. Conversely, MHC II expression was observed on dendritic or spindle-shaped to mononuclear cells, and its presence was correlated with the degree of mucosal inflammation. In tissues with a severity score of 0 to 1 (on a 0–3 scale), MHC II+ cells were few and scattered within the epithelium and lamina propria/submucosa, occasionally forming small, discrete clusters in the lamina propria or adjacent to skeletal muscle. Furthermore, some skeletal muscle fibers exhibited low-intensity, granular cytoplasmic expression of MHC II. In tissues with severity scores of 2 or 3, MHC II+ cells were found to be more numerous and densely packed, displaying variable morphology consistent with antigen-presenting cells (3).

The reviewed systemic characterization of the disease, identified ten exclusive biomarkers, including CD8^low^, CD21, CD45R, CD62L, total white blood cells (WBC), total neutrophils, total lymphocytes, total proteins (TP) and total albumin and globulins.

In the FCGS context, diseased animals exhibit elevated levels of CD8+ T cells, a reduced proportion of CD8^low^ cells, and a decreased CD4/CD8 ratio, due to the increased CD8+ T cells. The hypothesis was that the induction of CD8^low^ cells and the concurrent increase levels in IL6 may be responsible for the generation of T regulatory cells and the “reprogramming” of the immune response, resulting in a long-term cure in FCGS individuals. It is therefore possible to consider these two factors as biomarkers that predict the animal’s immune response (4).

The engagement of CD21 can modulate B cell responses by promoting survival, proliferation, and differentiation. Additionally, influences the development of memory B cells and long-lived plasma cells, which are crucial for mounting effective humoral immune responses upon re-exposure to pathogens (13, 53).

Similar to the above-mentioned biomarkers, individuals diagnosed with FCGS exhibited a markedly elevated WBC count compared to the control group, specifically caused by neutrophilia. This blood count, among others, is part of the Feline Life Stage Guideline (19), which should be carried out at all stages of a cat’s life. This features of systemic inflammation was observed to be longitudinally correlated with a number of other oral comorbidities (13, 19, 54).

CD62L, also known as L-selectin, is expressed on the surface of naive T cells and central memory T cells. It enables the migration of lymphocytes into secondary lymphoid organs, such as lymph nodes and Peyer’s patches. During asymmetric division, CD8+ T cells generate daughter cells with distinct properties, including different proteasome activities. Daughter cells with increased proteasome activity express memory CD8+ T cell biomarkers, including CD62L, IL-7R, and Tcf1. Pharmacological activation of the proteasome has been demonstrated to promote differentiation of memory CD8+ T cells. These cells can be effector memory (CD8+CD45-CD62L-) and central memory (CD8+CD45-CD62L+) cells. The most notable and consistent lymphocyte phenotype alteration observed in cats with FCGS was a significant increase in CD8+ effector memory cells CD8+CD45-CD62L-, accompanied by a reduction in central memory cells CD8+CD45-CD62L+. These CD8+ T cells also exhibited an activated phenotype, with increased percentages of CD25+ CD62L- cells (13, 44, 55).

Total proteins in blood plasma include albumin, globulins, and other proteins. These proteins play a variety of roles in immune function, including maintaining osmotic pressure, transporting nutrients and hormones, contributing to the immune response. Globulins include a few different types of proteins, including antibodies (immunoglobulins) produced by B cells, which play a crucial role in humoral immunity. The chemistry profile of FCGS individuals exhibits a high value of total protein (hyperglobulinemia), which is attributed to elevated levels of albumin, globulin, and monoclonal gammopathy (alpha 1 and 2, beta, and gamma 1 and gamma 2). A review of FCGS related studies indicates that systemic inflammation resulting from polyclonal hyperglobulinemia may serve as a potential biomarker for predicting treatment response, when compared to healthy and pre-treated individuals (7, 46, 56).

Biomarkers such as mast cells, plasma cells, CD3+ T cells, lymphocytes and neutrophils have been the focus of the majority of studies and reports in the local context, whereas cytokines such as IL-6, TNF-α, and IFN-γ, along with CD4 T cells, CD8 T cells and CD4/CD8+ ratio, have been more frequently reported systemically. This distribution underscores the dual nature of FCGS as a condition with both local and systemic immune dysregulation and emphasizes the necessity for comprehensive assessment in both compartments for diagnostic and therapeutic purposes.

## 4 Discussion

Systematic reviews are essential to evidence-based medicine and provide the strongest level of evidence to support decision-making. This type of study is distinguished by its methodological completeness, transparency, and replicability (57, 58).

FCGS presents a complex clinical and immunological challenge within the domain of veterinary medicine. The multifactorial nature of the disease, its variable response to treatment, and its immunopathological features highlight the necessity for a deeper understanding of its pathogenesis. The clinical manifestations of FCGS, such as gingivitis, alveolar stomatitis, and caudal stomatitis are widely reported in the literature (8, 9, 20, 24, 59) and closely mirror those bilateral and symmetrical manifestations observed in human OLP, where the caudal oral mucosa, gingiva, and lateral tongue margins are most frequently affected exhibiting a bilateral and symmetrical distribution (12).

Histopathological analysis is still considered a cornerstone in the diagnosing of FCGS, revealing lymphoplasmacytic infiltrates in the lamina propria and plasma cells in the subepithelial layers. This pattern is also characteristic of OLP (1, 3, 6, 27, 60). Immunohistochemical studies have demonstrated a predominance of CD3+ T lymphocytes in the epithelium and submucosa. In contrast, CD20+ B cells have been found to be mainly confined to the submucosal stroma (4, 6, 32). These observations suggest that FCGS may result from an aberrant immune response to persistent antigenic stimulation, possibly triggered by viral or bacterial agents. Nevertheless, infections with FIV, FeLV, FHV-1, and FCV have been isolated from both affected and clinically healthy cats (24, 61–63), thus challenging their direct etiological role.

Despite surgical interventions such as full-mouth extractions, approximately 30% of FCGS cases remain refractory (6, 32), and may require alternative treatment options (4, 8, 10, 27, 32). These animals often require lifelong immunomodulatory therapy, which is not without adverse effects, including polyuria, skin fragility, and secondary diabetes mellitus (6). Moreover, the financial implications and the potential for post-operative complications contribute to the complexity of treatment planning. Refractory cases are generally characterized by the absence of clinical improvement within 60 days following surgery (7), reinforcing the need for innovative, evidence-based therapeutic strategies (6, 64).

The immune dysregulation observed in FCGS includes both local and systemic alterations. In the specific local context, the chronic inflammatory response is characterized by the presence of mast cells, neutrophils, lymphocytes, macrophages, and plasma cells (65). Immunohistochemistry has been instrumental in identifying key players in the two main forms of immunity, humoral and cellular. These include immunoglobulins such as IgG, IgM, and IgA, as well as T and B cell subsets, including Mott cells (3, 27). Systemically, altered CD4/CD8 ratios, elevated neutrophil counts, and increased levels of IL-6, TNF-α, and IFN-γ are indicative of a heightened pro-inflammatory state and immune imbalance. The presence of reduced populations of CD21+ B cells and the dysregulation in regulatory T cell (Treg) subsets (FOXP3+) further emphasize the systemic nature of immune involvement.

This convergence between local and systemic immune activity suggests a mechanistic link, in which chronic mucosal inflammation in FCGS may not only reflect localized immune dysfunction but also act as a trigger for systemic immune activation. The existence of such overlap underscores FCGS as a condition with both localized and systemic immunological consequences, thereby suggesting a pathophysiological association.

A comparative immunopathology analysis highlights striking parallels between FCGS and human OLP. Both diseases are characterized by chronic T cell-driven inflammation, dominated by CD4+ helper and CD8+ cytotoxic T cells, along with Treg infiltration (14, 47). However, in both conditions, Tregs (indicated by CD25 and FOXP3 expression) appear to be functionally impaired despite being present, as evidenced by decreased suppressive capacity and co-expression of pro-inflammatory biomarkers such as IL-17 in OLP (66, 67). This phenotypic instability is likely to contribute to the persistence of inflammation and resistance to therapy in both diseases. The cytokine profiles are similarly aligned, with increased IL-6, TNF-α, and IFN-γ levels in affected tissues. In contrast, elevated levels have been detected systemically in both serum and saliva in FCGS cases (66, 68).

Based on the clinical presentation and immune response mechanisms, it is proposed that, FCGS involves the activation of plasmacytoid dendritic cells, being a central feature. In human OLP, this activation results in the production of IFN-α by dendritic cells and other cytokines, promoting antigen presentation to CD4+ and CD8+ T-lymphocytes. The co-expression of CD40 and CD80, in combination with IL-12 secretion by dendritic cells, triggers a CD4+ Th1 response. Subsequent interactions involve the release of IL-2 and IFN-γ by CD4+ Th1 cells, which bind to receptors on cytotoxic CD8+ lymphocytes (47). Upon activation, these cells have been observed to induce the secretion of granzyme/perforin and/or TNF-α. Non-specific mechanisms, such as mast cell degranulation and metalloproteinase activation, contribute to the disruption of the basement membrane, which facilitates the intraepithelial migration of T-lymphocytes (50). It is hypothesized that the CD8^low^ T cells subset may have distinct functional properties compared to the conventional CD8+ T cells (45). The perpetuation of the inflammatory response remains to be fully elucidated, although it may involve failures in the suppression of inflammation, such as the reduced apoptosis of cytotoxic T-lymphocytes and the decreased inhibition of proinflammatory cytokines (12).

Additionally, while drug-induced gingivostomatitis is recognized in human OLP using antihypertensives, non-steroidal anti-inflammatory drugs (NSAIDs), anti-infectious agents, psychotropic/neuroleptic agents, hypoglycemic agents, antimycotics, heavy metals and others such as allopurinol and iodinated contrast agents, there are no equivalent reports in veterinary literature regarding FCGS (67). Nonetheless, both conditions are considered to be immune-mediated diseases influenced by a combination of genetic, hormonal, microbial, nutritional, and environmental factors, including stress and mechanical trauma (16).

A critical distinction in immunological understanding involves the differentiation between ‘oral tolerance’ and ‘oral mucosal tolerance’. The term ‘oral tolerance’ refers to a state of systemic hyporesponsiveness induced by antigen exposure via the gastrointestinal tract, and this process is mediated by gut-associated lymphoid tissue. In contrast, ‘oral mucosal tolerance’ constitutes a localized immune regulatory mechanism initiated within the oral mucosa through antigen presentation by resident dendritic cells and macrophage-like cells (50, 65, 69). This distinction is essential when interpreting mucosal immune responses in diseases like FCGS.

The parallels between FCGS and OLP extend beyond clinical, immunopathological and histopathological features, supporting the use of FCGS as a spontaneous translational model for investigating mucosal immune dysregulation and chronic inflammatory responses relevant to human autoimmune diseases. The chronic nature of both diseases, which is possibly associated with ongoing antigenic stimulation, is well documented, as are the relapses and resistance to conventional treatment that frequently require long-term immunotherapy. It is therefore evident that they function as significant models for the exploration of immune homeostasis breakdown and the development of targeted therapies.

Despite the insights gained from this review, significant knowledge gaps remain, particularly concerning the etiopathogenesis and immune mechanisms underlying FCGS. It is recommended that future research place a priority on the standardization of diagnostic and staging criteria, in addition to the design of robust clinical trials that poses adequate sample sizes and statistical power. Such efforts are essential to advance individualized treatment strategies grounded in a comprehensive understanding of local and systemic immune responses.

This systematic review makes a substantial contribution to the field of veterinary immunopathology by consolidating the current knowledge on FCGS and identifying shared features with human OLP. The study provides a foundation for further exploration of immune-targeted therapies and reinforces the importance of translational approaches in managing complex oral inflammatory diseases.

## Supplementary material

Supplementary Table 1 Table with the summary of the diagnostic methods and biomarkers used to characterize the immune local presentation in FCGS, as presented in the 17 selected articles. HP, Histopathology; IHC, Immunohistochemistry; NM, Not Mentioned; PCR, Polymerase Chain Reaction.

Supplementary Table 2 Table with the summary of the laboratorial methods, sample types, and biomarkers used to characterize systemic presentation of FCGS, as presented in the 17 selected articles. ELISA, Enzyme-Linked Immunosorbent Assay; NM, Not Mentioned; PBMC, Peripheral Blood Mononuclear Cell; WBC, White Blood Cells.
