# Supplementary material for: Local and systemic immunological response in feline chronic gingivostomatitis: a critical review
Source: Front Immunol. 2025 Sep 11;16:1572631. doi: 10.3389/fimmu.2025.1572631 (PMC12460127; doi:10.3389/fimmu.2025.1572631)
Supplement: Supplementary Table 1 — Table with the summary of the diagnostic methods and biomarkers used to characterize the immune local presentation in FCGS, as presented in the 17 selected articles. HP, Histopathology; IHC, Immunohistochemistry; NM, Not Mentioned; PCR, Polymerase Chain Reaction. [file DataSheet1.pdf]

Supplementary Table 1:

Summary of the diagnostic methods and biomarkers used to characterise the local immunology of FCGS, as presented in the 17 selected articles. Legend: HP – Histopathology, IHC – Immunohistochemistry, NM – Not Mentioned, PCR – Polymerase Chain Reaction.

Diagnostic method	Laboratorial technique	Biomarkers/cells	References
Clinical and Laboratorial	HP	Lymphocytes; Neutrophils; Macrophages; Plasma cells	Hennet, 1997
Clinical and Laboratorial	NM	Salivary IgG; IgM; IgA; Albumin	Harley et al., 2003
Clinical	NM	NM	Healey et al., 2007
Clinical and Laboratorial	HP	Lymphocytes; Plasma cells	Dowers et al., 2009
Clinical and Laboratorial	HP	Lymphocytes; Neutrophils; Macrophages; Plasma cells; Mast cells	Arzi et al., 2010
Clinical and Laboratorial	HP + IHC	CD3; CD4; CD8; CD79α; IgG; IgM; IgA; MHC II; Plasma cells; L1; Mast cells	Harley et al., 2011
Clinical and Laboratorial	PCR	TLR2; TLR3; TLR4; TLR7; TLR9; IL-1β; IL-4; IL-6; IL-10; IL-12; TNF-α; INF-γ	Dolieslager et al., 2013
Clinical and Laboratorial	HP + IHC	CD3; CD20; Lymphocytes; Neutrophils; Plasma cells; Mott cells; macrophages; Mast cells	Arzi et al., 2016
Clinical and Laboratorial	HP + IHC	CD3; CD20; Lymphocytes; Neutrophils; Plasma cells; Mott cells; Macrophages; Mast cells	Arzi et al., 2017
Clinical	NM	NM	Druet et al., 2017
Clinical and Laboratorial	HP + IHC	Lymphocytes; Neutrophils; Plasma cells; Mott cells; Mast cells	Rolim et al., 2017
Laboratorial	HP + IHC	CD3; CD79α; S100; Mast cells	Mikiewicz et al., 2019
Clinical and Laboratorial	HP + IHC	CD3; CD4; CD8; CD20; CD25; FoxP3; Lymphocytes; Neutrophils; Macrophages; Plasma cells; Mott cells; Mast cells; Dendritic Cells	Vapniarsky et al., 2020
Clinical and Laboratorial	HP + IHC	CD3; CD20; Lymphocytes; Neutrophils; Plasma cells; Mott cells; Macrophages; Mast cells	Arzi et al., 2020
Clinical and Laboratorial	HP	NM	Fried et al., 2020
Clinical and Laboratorial	IHC	NM	Peralta et al., 2023
Clinical	NM	NM	Fontes et al., 2023
