# Supplementary material for: Local and systemic immunological response in feline chronic gingivostomatitis: a critical review
Source: Front Immunol. 2025 Sep 11;16:1572631. doi: 10.3389/fimmu.2025.1572631 (PMC12460127; doi:10.3389/fimmu.2025.1572631)
Supplement: Supplementary Table 2 — Table with the summary of the laboratorial methods, sample types, and biomarkers used to characterize systemic presentation of FCGS, as presented in the 17 selected articles. ELISA, Enzyme-Linked Immunosorbent Assay; NM, Not Mentioned; PBMC, Peripheral Blood Mononuclear Cell; WBC, White Blood Cells. [file DataSheet2.pdf]

Supplementary Table 2:

Summary of the laboratorial methods, biological material and studied biomarkers used to characterise the systemic features of FCGS, as presented in the 17 selected articles. Legend: ELISA - Enzyme-Linked Immunosorbent Assay, NM – Not Mentioned, PBMC - Peripheral Blood Mononuclear Cell, WBC - White Blood Cells.

Method	Biological material	Biomarkers	References
NM	NM	NM	Hennet, 1997
ELISA	Serum	IgG; IgM; IgA	Harley et al., 2003
NM	NM	NM	Healey et al., 2007
NM	NM	NM	Dowers et al., 2009
NM	NM	NM	Arzi et al., 2010
NM	NM	NM	Harley et al., 2011
NM	NM	NM	Dolieslager et al., 2013
Flow Cytometry + Blood cell count + ELISA + Electrophoresis	Total blood, Serum	CD4; CD8; CD8lo; CD4/CD8 ratio; IgA; IgG; IL-1β; IL-6; TNF-α; INF-γ; Globulins; Total WBC; Neutrophils; Lymphocytes	Arzi et al., 2016
Flow Cytometry + Blood cell count + ELISA + Electrophoresis	Total blood, Serum	CD4; CD8; CD8lo; CD4/CD8 ratio; IL-6; TNF-α; INF-γ; Globulins; Total WBC; Neutrophils, PBMC	Arzi et al., 2017
NM	NM	NM	Druet et al., 2017
NM	NM	NM	Rolim et al., 2017
NM	NM	NM	Mikiewicz et al., 2019
Flow Cytometry + Blood cell count + Serum biochemistry	Total blood, Serum	CD3; CD4; CD8; CD4/CD8 ratio; CD21; CD25; CD45R; CD62L; FoxP3; Total WBC; Neutrophils; Lymphocytes, PBMC	Vapniarsky et al., 2020

Flow Cytometry + Blood cell count + Serum biochemistry	Total blood, Serum	CD4; CD8; CD8lo; CD4/CD8 ratio; CD21; Total proteins; Albumin; Globulins; Total WBC; Neutrophils; Lymphocytes, PBMC	Arzi et al., 2020
NM	NM	NM	Fried et al., 2020
NM	NM	NM	Peralta et al., 2023
NM	NM	NM	Fontes et al., 2023
